# Identification of key regulators in Sarcoidosis through multidimensional systems biological approach

**DOI:** 10.1038/s41598-022-05129-7

**Published:** 2022-01-24

**Authors:** Safia Tazyeen, Mohd Murshad Ahmed, Anam Farooqui, Aftab Alam, Md. Zubbair Malik, Mohd Saeed, Irfan Ahmad, Mohammed Abohashrh, R. K. Brojen Singh, Romana Ishrat

**Affiliations:** 1 Centre for Interdisciplinary Research in Basic Science, Jamia Millia Islamia, New Delhi, 110025 India; 2 School of Computational and Integrative Sciences, Jawaharlal Nehru University, New Delhi, 110067 India; 3 Department of Biology, College of Sciences, University of Hail, Hail, 2440 Saudi Arabia; 4 Department of Clinical Laboratory Science, College of Applied Medical Sciences, King Khalid University, Abha, 61421 Saudi Arabia; 5 Research Center for Advanced Materials Science, King Khalid University, Abha, 61421 Saudi Arabia; 6 Department of Basic Medical Sciences, College of Applied Medical Sciences, King Khalid University, Abha, 61421 Saudi Arabia

**Keywords:** Computational biology and bioinformatics, Biomarkers

## Abstract

Sarcoidosis is a multi-organ disorder where immunology, genetic and environmental factors play a key role in causing Sarcoidosis, but its molecular mechanism remains unclear. Identification of its genetics profiling that regulates the Sarcoidosis network will be one of the main challenges to understand its aetiology. We have identified differentially expressed genes (DEGs) by analyzing the gene expression profiling of Sarcoidosis and compared it with healthy control. Gene set enrichment analysis showed that these DEGs were mainly enriched in the inflammatory response, immune system, and pathways in cancer. Sarcoidosis protein interaction network was constructed by a total of 877 DEGs (up-down) and calculated its network topological properties, which follow hierarchical scale-free fractal nature up to six levels of the organization. We identified a large number of leading hubs that contain six key regulators (KRs) including ICOS, CTLA4, FLT3LG, CD33, GPR29 and ITGA4 are deeply rooted in the network from top to bottom, considering a backbone of the network. We identified the transcriptional factors (TFs) which are closely interacted with KRs. These genes and their TFs regulating the Sarcoidosis network are expected to be the main target for the therapeutic approaches and potential biomarkers. However, experimental validations of KRs needed to confirm their efficacy.

## Introduction

Sarcoidosis (SARC) is an inflammatory disease (multiple organ inflammation) that causes abnormal granulomas consisting of inflamed tissue that is usually observed in the lungs and lymph glands. These granulomas may alter the normal structure and function of the affected organs. SARC affects people of all ages, genders, and ethnic backgrounds. It usually affects adults less than 40 years of age, and the incidence peaks in the third decade of life and is less common in children. Many studies reported a slightly higher rate of incidence in women across racial/ethnic groups^1^. The worldwide prevalence varies from 2 to 80 per 100,000^2^. In India, the prevalence is estimated to be 10–12 per 1000^3^. However, in 30–60% of the cases the prevalence may be underestimated by the asymptomatic signs of the disease. In Afro-Americans, the incidence is three times higher as compared to Caucasians and it is also more likely to be fatal^4^. The understanding of SARC has been challenging because of the multiple issues. Clinically, SARC is extremely complex because patients do not typically exhibit clear signs and symptoms; it varies depending on the organ affected.

In the present era, the research on SARC has been focused on its pathological mechanism.It is believed that when a genetically susceptible individual is exposed to one or more extrinsic antigens, inflammatory pathways are over-activated, favoring the formation of sarcoidal granulomas. It has been suggested that there is an increased risk of SARC in individuals exposed to environmental entities such as microbial agents etc.^4^. Susceptibility to the disease can be genetically determined and many genes have been identified that affect the prevalence and course of SARC. In particular, HLA genes have been shown to affect the progression of SARC and its development^5^. Cytokines like interferon-gamma (IFN-γ), IL-12 and TNF-α have been involved in the SARC formation^6^. The identified causes include inflammation, genetic polymorphism, and development of granulomas and so on, yet SARC's primary causes and the vast majority of involved genes are still unclear.

High throughput technology, such as microarray, has facilitated research to discover new pathogenic SARC mechanisms. Significant quantities of information, specifically regarding the microarray-based mRNA expression analysis of pathological tissue including lymph nodes, blood cells, and lungs^6,7^. Separately, bioinformatics analysis of gene expression analysis can identify highly regulated molecular pathways which are likely to enhance abnormal granulomatous inflammation. Upregulated VEGF and HIF1A genes have been associated with recognized negative prognostics^8^. MiRNAs and Transcription Factors (TFs) are two types of essential gene regulators that participate in many fundamental cellular processes and have a common regulatory logic in the co-regulation of target genes, among several other genetic factors. TFs affect gene transcription at the transcriptional level, whereas MiRNAs primarily regulate gene expression at the post-transcriptional level. Furthermore, as gene regulators, how miRNAs and TFs work together to regulate gene expression to induce SARC pathogenesis has yet to be studied.

Genes are regulated in a coordinated fashion, and the absence or presence of another gene normally depends on the expression of one gene (i.e., gene interaction). The network theory is an imperative approach for understanding the dynamics and properties of complex regulatory networks. The network’s small world, scale-free, random and hierarchical nature falls mostly within a real network. The hierarchical network is of particular interest to the biologist because it integrates modules, sparsely dispersed hubs that regulate the network and its self-organizing working concept. A recent study on the complex protein–protein interaction (PPI) network suggests its conformity to scale-free topology on a hierarchical scale^9^. On these networks, the problem arises that the central lethality rule does not apply where the stability and dynamics of the network are disrupted but not completely disrupted when the hubs are targeted^9^. This may be due to the hierarchical organization of community/sub-communities in complex networks and other biological networks at various topological levels, where specific roles are associated with them^10–12^.

In this study, the DEGs were analyzed by microarray expression profiles based on the GEO datasets between Sarcoidosis and healthy control. The biological function and pathway enrichment analysis were also performed. SARC network was constructed from DEGs (up-down) and then analyzed its topological properties from which we are trying to predict potential key regulators among them some of having its fundamental importance of regulating as well as their activities mechanism. Further, we identified hubs, community/modules and sub-communities which control the network stability as well as other communities. Additionally, to assess the interactions between the transcription factors and key regulators, a gene-TFs regulation network of key regulators was also assessed. The findings of this study are expected to increase our understanding of the genes or proteins involved in the formation and development of SARC, which will support the various therapeutic approaches for Sarcoidosis.

## Results

### Gene expression profiling of sarcoidosis through microarray data

This study provides information on the structure of correlation-based tuning between genes in multiple microarray datasets by comparing analysis across datasets that is relevant in understanding gene functions. Each series has a different number of differentially expression genes, as presented in Table 1. Based on the differential expression analysis of six GSE series, a total of 1,182 DEGs were identified, of which 263 were up-regulated and 919 were down-regulated genes, respectively (Table S1).

**Table 1:** Detailed information on the Gene expression microarray datasets related to Sarcoidosis.

GEO accession	Platform	No. of probes	Experiment type	No. of samples (controls/disease)	Samples types	Log fold change	DEGs (up/down)
GSE16538	GPL570	54,675	Expression profiling by array	12 samples (6/6)	Lung biopsy	≥ 1 and ≤ − 1	32/198
GSE18781	GPL570	54,675	Expression profiling by array	37 samples (25/12)	peripheral blood	≥ 1 and ≤ − 1	104/75
GSE19314	GPL570	54,675	Expression profiling by array	58 samples (20/38)	peripheral blood mononuclear cells	≥ 1 and ≤ − 1	16/16
GSE19976	GPL6244	33,297	Expression profiling by array	15 samples (8/7)	Lung biopsy	≥ 1 and ≤ − 1	55/342
GSE37912	GPL5175	21,788	Expression profiling by array	74 samples (35/39)	peripheral blood mononuclear cells	≥ 1 and ≤ − 1	15/18
GSE75023	GPL571	22,277	Expression profiling by array	27 samples (12/15)	Bronchoalveolar cells	≥ 1 and ≤ − 1	48/207

### Gene ontology and pathway analysis of DEGs

The biological function and pathways enrichment was analyzed for a total of 172 up and 705 down-regulated genes. We found that the DEGs were significantly enriched in many biological, cellular, and molecular functions as well as some pathways. The modified Fisher exact *p*-value (EASE score) ≤ 0.05 is considered strongly enriched. The top 10 enriched biological functions are presented in Table 2. By analyzing the BP, we found that the up-regulated DEGs from the SARC's PPI network were enriched in positive regulation of gene expression, positive regulation of protein kinase activity, osteoblast differentiation, inflammatory response, and single organismal cell–cell adhesion. At the same time, the down-regulated DEGs were significantly involved in the immune response, inflammatory response, signal transduction, adaptive immune response, and innate immune response. The up-regulated DEGs were correlated with the plasma membrane, an integral component of the plasma membrane, Golgi apparatus, extracellular exosomes, and cell surface for the CC analysis. In contrast, the down-regulated DEGs were linked with the integral component of the plasma membrane, external side of the plasma membrane, cell surface, extracellular region, and plasma membrane. The up-regulated DEGs were enriched in translation initiation factor activity, transporter activity, protein binding, very-low-density lipoprotein particle receptor, and manganese ion trans- membrane transporter activity for the MF analysis. In contrast, the down-regulated DEGs were related to receptor binding, receptor activity, actin-binding, trans-membrane signaling receptor activity, and protein binding. For KEGG pathways enrichment analysis, the up-regulated DEGs were not enriched. In contrast, the 5 top significant KEGG pathways of the down-regulated DEGs included cytokine-cytokine receptor interaction, Tuberculosis, Osteoclast differentiation, pathways in cancer, and Human T lymphotropic virus type I (HTLVI) infection.

**Table 2:** The gene ontology and pathway enrichment of DEGs of sarcoidosis.

Category	Term	Count		P-value
**Up regulated DEGs**				
BP	Positive regulation of gene expression	10		8.89E−04
	Positive regulation of protein kinase activity	5		0.001006
	Osteoblast differentiation	6		0.003096
	Manganese ion transport	3		0.005525
	Protein kinase C signaling	3		0.0098
	Inflammatory response	10		0.010158
	Single organismal cell–cell adhesion	5		0.015548
	Positive regulation of humoral immune response	2		0.02814
	Positive regulation of phagocytosis	3		0.030605
	Iron ion homeostasis	3		0.032591
CC	Plasma membrane	53		0.005109
	Extracellular exosome	39		0.006336
	Integral component of plasma membrane	23		0.008882
	Extracellular space	22		0.010231
	Golgi apparatus	16		0.011843
	Clathrin-coated pit	4		0.013554
	Extrinsic component of cytoplasmic side of plasma membrane	4		0.023785
	Cell surface	11		0.025944
	Postsynaptic density	6		0.026169
	Viral nucleocapsid	3		0.026401
MF	Protein binding	97		0.006188
	Translation initiation factor activity	4		0.01822
	Manganese ion transmembrane transporter activity	2		0.027121
	Very-low-density lipoprotein particle receptor activity	2		0.035998
	Transporter activity	6		0.037743
**Down regulated DEGs**				
BP	Immune response	91		3.31E−42
	Inflammatory response	75		5.81E−32
	Adaptive immune response	38		2.63E−20
	Signal transduction	106		4.90E−17
	Innate immune response	57		8.68E−16
	Cell surface receptor signaling pathway	44		2.88E−15
	Positive regulation of GTPase activity	48		5.60E−07
	Cell adhesion	40		3.09E−06
	Apoptotic process	41		1.64E−04
	G-protein coupled receptor signaling pathway	48		0.019548
CC	Integral component of plasma membrane	126		5.26E−21
	External side of plasma membrane	43		1.95E−19
	Plasma membrane	240		2.56E−15
	Membrane	145		1.28E−12
	Extracellular space	100		1.58E−11
	Cell surface	55		2.54E−11
	Extracellular region	97		1.17E−06
	Integral component of membrane	243		3.90E−06
	Extracellular exosome	144		1.79E−05
	Cytosol	159		1.84E−04
MF	Receptor activity	34		8.17E−12
	Transmembrane signaling receptor activity	28		4.04E−08
	Receptor binding	36		2.01E−07
	Carbohydrate binding	24		1.48E−06
	Protein binding	389		3.36E−06
	Cytokine activity	20		3.89E−05
	Protein homodimerization activity	45		0.001576
	Actin binding	22		0.002086
	Protein kinase binding	24		0.016353
	Chemokine activity	19		7.55E−14
	Cytokine–cytokine receptor interaction	62		6.31E−21
KEGG	Pathways in cancer	37		0.027273
	Tuberculosis	39		4.51E−11
	HTLV-I infection	39		1.36E−06
	Osteoclast differentiation	41		3.13E−17
	Chemokine signaling pathway	52		2.05E−19
	Cell adhesion molecules (CAMs)	34		9.82E−11
	Phagosome	31		3.06E−08
	Hematopoietic cell lineage	30		7.34E−14
	Rheumatoid arthritis	30		1.03E−13

### SARC network: hierarchical scale-free features

**Figure 1:**
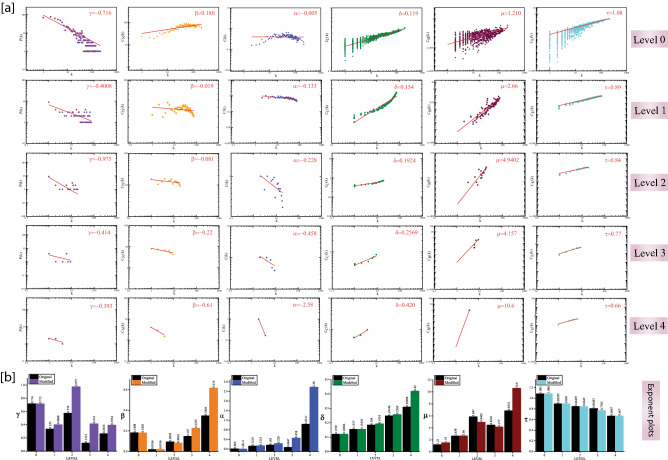
SARC PPI network and sub-networks followed hierarchical scale-free topologies. (**a**) The behaviors of degree distributions P(k), neighborhood connectivity C_N_(k), clustering co-efficient (k), closeness C_C_(k), betweenness C_B_(k) and eigenvector C_E_(k) measurements as a function with degree k for an original primary network (level 0) and FLT3LG-CD33-ITGA4 motif knockout networks at various levels of organization (level 1–4). (**b**) the changes in the exponent values of the six topological properties of the FLT3LG-CD33-ITGA4 motif knockout network [colors corresponding to the ones used in the topological properties plots, i.e., violet for P(k), orange for C_N_(k), blue for C(k), green for C_C_(k), maroon for C_B_(k) and cyan for C_E_(k)] compared with the topological properties’ exponents of the corresponding original networks (black) at various levels of the organization. γ, α, β, δ, µ and τ are the exponents of the degree distribution, neighborhood connectivity, clustering coefficient, closeness centrality, betweenness centrality and eigenvector centrality, respectively.

**Figure 2:**
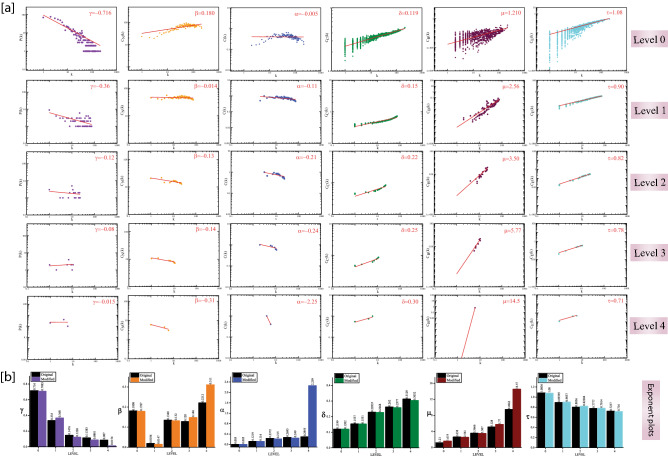
SARC PPI network and sub-networks followed hierarchical scale-free topologies. (**a**) The behaviors of degree distributions P(k), neighborhood connectivity C_N_(k), clustering co-efficient (k), closeness C_C_(k), betweenness C_B_(k) and eigenvector C_E_(k) measurements as a function with degree k for an original primary network (level 0) and ICOS-CTLA4-GPR29 motif knockout networks at various levels of organization (level 1–4). (**b**) the changes in the exponent values of the six topological properties of the ICOS-CTLA4-GPR29 motif knockout network [colors corresponding to the ones used in the topological properties plots, i.e., violet for P(k), orange for C_N_(k), blue for C(k), green for C_C_(k), maroon for C_B_(k) and cyan for C_E_(k)] compared with the topological properties’ exponents of the corresponding original networks (black) at various levels of the organization. γ, α, β, δ, µ and τ are the exponents of the degree distribution, neighborhood connectivity, clustering coefficient, closeness centrality, betweenness centrality and eigenvector centrality, respectively.

The primary SARC PPI network was constructed by up and down-regulated genes that contain 877 nodes and 10,546 edges; the remaining genes have not shown their interaction and were excluded from the network. The network's topological properties follow hierarchical characteristics^13^ and scale-free behavior in these parameters because of the power-law nature^14,15^. The probability of node degree distributions (P), clustering coefficient (C), and neighborhood connectivity (CN) against degree k exhibit fractal nature or power-law (Figs. 1a, 2a first rows against level 0).

The power-law fits on the data distributions was performed and validated by following the standard statistical fitting procedure given by Clauset et al*.*^16^, where, all the statistical p-value for all datasets was calculated against 2500 random sampling are found to be > 0.1 (greater than one), and the goodness of fits are found to be ≤ 0.33 (less than and equal to) which is the threshold value predicted. These distributions are done on a log–log plot through a straight line^15^.

1 $$\left(\begin{array}{c}P\\ C\\ {C}_{N}\end{array}\right) \sim \left(\begin{array}{c}{K}^{-\gamma }\\ {K}^{-\alpha }\\ {K}^{+\beta }\end{array}\right);\left(\begin{array}{c}{\gamma }_{0}\\ {\alpha }_{0}\\ {\beta }_{0}\end{array}\right) \to \left(\begin{array}{c}0.716\\ 0.005\\ 0.180\end{array}\right)$$

The negative value of P(k) and C(k) indicates that the primary SARC network follows a hierarchical scale-free fractal network. The positive value of C_N_(k) indicates the nature of assortativity that regulates the primary SARC network by identifying a large cluster of degree-nodes (rich club formation).

2 $$\left(\begin{array}{c}{C}_{C}\\ {C}_{B}\\ {C}_{E}\end{array}\right) \sim \left(\begin{array}{c}{K}^{\delta }\\ {K}^{\mu }\\ {K}^{\tau }\end{array}\right);\left(\begin{array}{c}{\delta }_{0}\\ {\mu }_{0}\\ {\tau }_{0}\end{array}\right) \to \left(\begin{array}{c}0.119\\ 1.210\\ 1.083\end{array}\right)$$

Similarly, the network centrality parameters: closeness (C_C_), betweenness (C_B_), and eigenvector (C_E_) centralities also show fractal behavior. The positive values of these centrality parameters indicate that the leading hubs in the SARC network play a strong regulatory role.

### Key regulators and properties of SARC network

**Figure 3:**
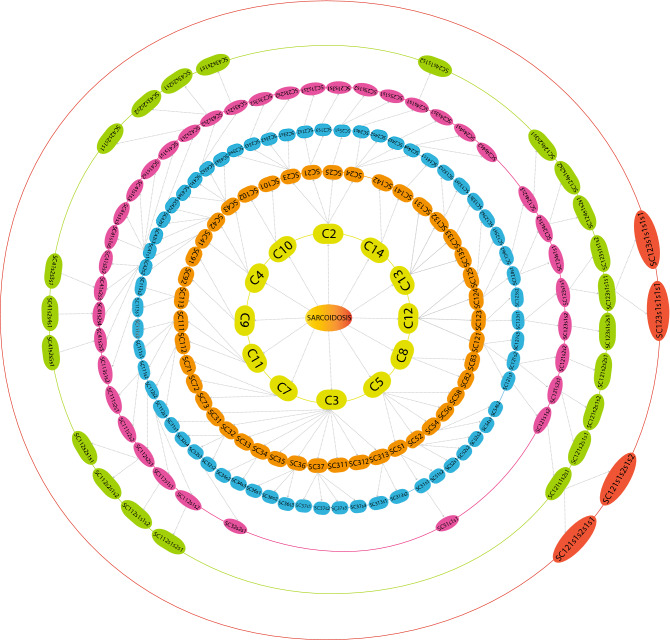
Representation of the organization of the SARC network/communities/sub-communities at six various levels, and arrows indicate sub-communities constructed from the previous community.

**Figure 4:**
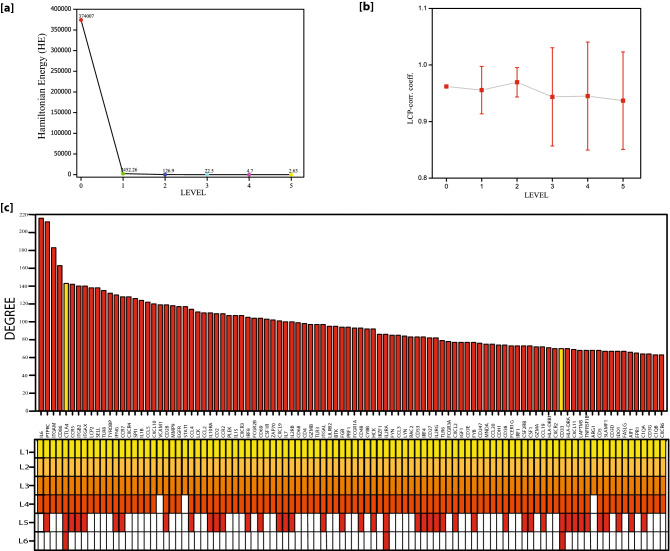
(**a**) Plots of Hamiltonian Energy as a function of level of organization. (**b**) Plots of LCP-corr as a function of the level of organization. (**c**) Characterization of top hundred leading hubs in the complete network by degrees; the plot also indicates unpredictability of these leading hubs at various levels of the organization. CTLA4 and CD33 are the key regulators presented by yellow color.

In the SARC Network, we have found fifteen communities that were further broken down into sub-community and sub-sub-community up to sixth level. The modular structure and its arrangement were carried out by the standard community finding techniques of Newman and Girvan^17^ at different organizational levels (Fig. 3). Using this approach, we found that our network is organized hierarchically through six different levels. The corresponding Hamiltonian Energy (HE) is decreased from top to bottom in a network organization against the different organizational levels (Fig. 4a).

The leading hubs (nodes) are essential regulators depending on the changes in the activities of proteins/genes and their regulating mechanism. All of the leading hubs are not a key regulator for disease progression, but only those hubs that regulate the network from top to bottom where the network cannot be further divided into sub-community and form motif have been considered to be important leading hubs. We termed them as "Key Regulators (KRs)" because; they were deeply rooted hub genes which can reach motif level (fundamental regulating unit) through different levels of the organization via communities or sub-communities from primary network to motif level. These key regulators are treated as the backbone to maintaining the network’s stability, as they capacitate the network to tackle any unacceptable changes in it.

We identified six key regulators, namely ICOS, CTLA4, GPR29, FLT3LG, CD33, and ITGA4, which are the SARC network's key regulators or organizers. These key regulators were separated from each other after level 2, *ICOS-CTLA4-GPR29* moved into the same sub-communities, and *FLT3LG-CD33-ITGA4* moved into another sub-community and then moved separately till the sixth level (motif). *ICOS-CTLA4-GPR29* and *FLT3LG*-CD33-*ITGA4* are forming a triangular motif (Fig. 6a). *ANPEP-IL2RA* and *FOXN2-TNFR3F25* reached the sixth level but because they don’t form motif they could not be considered as key regulators (Fig. S1).

Then, the top 100 hubs were ranked by the number of degrees. Surprisingly, none of these KRs genes fall into the top 10 leading hubs categories. However, two key regulators, C*TLA4* and *CD33* were among the top 100 high degree hubs (Fig. 4c). It means that KRs don’t always need to be the network's large leading hubs; rather, they can randomly change their popularity at various levels of an organization (Fig. 3). All the key regulators maintained low popularity or profile, but essential regulators in the SARC network; they regulate the motif level of organization. Few more genes, namely, *ANPEP, IL2RA*, *FOXN2 and TNFR3F25* supported the network reached till the sixth level. *IL2RA* was among the top 100 high degree hub genes. These key regulators act as signal propagators from top to bottom and vice versa to maintain the stability of the networks, whenever the network is under external stress and inherent properties.

**Figure 5:**
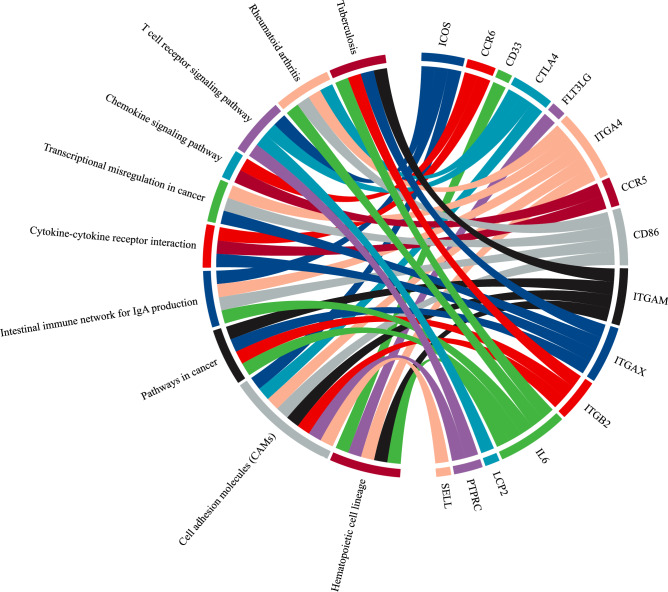
Chord plot showing the association between significantly enriched pathways and the top 10 leading hubs and key regulators involved. The outer circle indicates the top 10 significantly enriched pathways (on the left) and the top 10 leading hubs and key regulators (on the right). Each gene has a different color band, and the undirected colored edge inside the circle represents the relationship of a particular gene with their respective connected pathway(s).

According to the highest degree, the top 10 leading hubs are IL6, PTPRC, ITGAM, CD86, CTLA4, CCR5, ITGB2, ITGAX, LCP2 and SELL. Functional pathways enrichment analysis suggested that the top 10 leading hub and key regulators are mainly enriched in the Hematopoietic cell lineage, Cell adhesion molecules (CAMs), Pathways in cancer, Intestinal immune network for IgA production, Tuberculosis, Transcriptional misregulation in cancer, Rheumatoid arthritis, T cell receptor signaling pathway, Chemokine signaling pathway and Cytokine-cytokine receptor interaction (Fig. 5).

We have computed the Probability ${P}_{y}\left({x}^{l}\right)$ of key regulators to understand the regulating ability of each of the six key regulators:

3 $${{\varvec{P}}}_{{\varvec{y}}}\left({{\varvec{x}}}^{\left[{\varvec{l}}\right]}\right)=\boldsymbol{ }\frac{{{\varvec{x}}}^{\left[{\varvec{l}}\right]}}{{{\varvec{E}}}^{\left[{\varvec{l}}\right]}}$$

where, x = number of edges x^*[l]*^ at level *l*. *E*^*[l]*^ = total number of edges of the network or modules or sub-modules.

**Figure 6:**
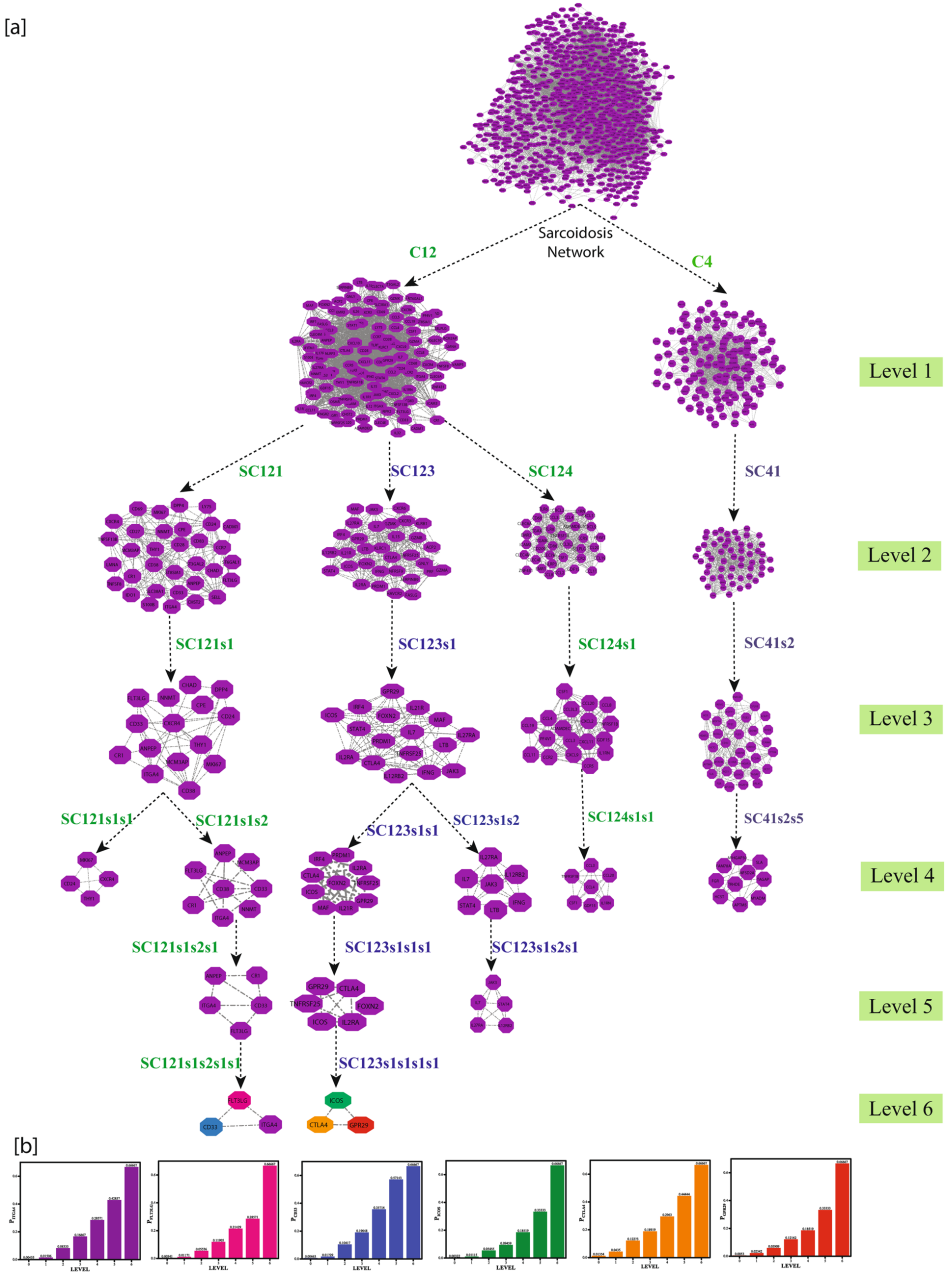
(**a**) The modular path of key regulators starting from the primary network to the motif levels. (**b**) The probability distribution of the key regulators as a function of the level.

The computed Probability ${P}_{y}\left({x}^{l}\right)$ of all the key regulators shows an increase in ${P}_{y}$ values from top to bottom, which increases the level *l.* This means the regulatory role of each fundamental regulator becomes more powerful at deeper levels of the organization and active workers at the grassroots level (Fig. 6b).

### Key regulators knock out experiments

In order to understand the organization, re-organization and significance of the key regulators in a SARC network, changes in the topological properties of the network are finally studied by removing key regulators from the network. It demonstrates the importance of the key regulators in the SARC Network. The knockout experiment was carried out separately for both the motifs; *FLT3LG-CD33-ITGA4* and *ICOS-CTLA4-GPR29* are triangular motifs. In both cases, a considerable change in the topological properties of the network has been observed, but somehow the network was reorganized itself and has tolerance against network error.

In all the key regulators or motif knockout network, the decrease in the exponent of P(k) *γ* indicates that the network self-reorganizes to stabilize and save the network properties from the breakdown. The increase in exponent of C(k) *α* indicates community compactness increases to save the communities from breakdown. In the deeper levels of the organization, the positive exponent value of C_N_ (k) β becomes negative, which indicates that the network is most tolerant and dis-assortative in nature. It is observed that the exponent value of C_B_(k)* μ* in the network first increases then decreases because of the removal of key regulators but again, the value of *μ* increases, which indicates the decreasing importance of the regulatory roles of the remaining hubs but reorganize themselves to prevent the network breakdown. The increase in exponent of C_C_(k) δ indicates that information processing in the network becomes faster when key regulators are removed, and hence reorganize the perturbed network and save it from breakdown. Further, the decreases in the exponent value of eigenvector centrality τ indicate that transmission of information is diminished because the key regulators are removed (Figs. 1a, 2a). In all the key regulators or motif knockout experiments, the values of the exponent for all the topological properties show drastic changes in deeper levels of the organization, but we did not get a breakdown of the network and maintains the hierarchical features of its organization after removing the key regulators or motif (Figs. 1b, 2b).

The change in γ etc., for Figs. 1b and 2b gives an overall picture of how important these two motifs. While ICOS-CTLA4-GPR29 motif knockout has greater impact on destroying scale free and assortative nature of the network at lower levels, on the other hand FLT3LG-CD33-ITGA4 motif has a little or no effect on the integrity of the network as compared to ICOS-CTLA4-GPR29 motif.

### Energy distribution in the network: calculation of Hamiltonian energy

**Figure 7:**
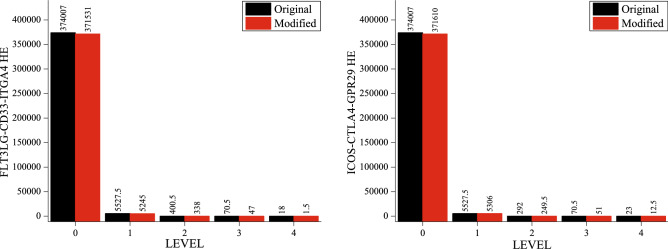
Comparison to the Hamiltonian energy of the original (black) and the corresponding key regulators knockout network (red) at various levels of organization of the SARC network.

The Hamiltonian Energy calculations for a network within CPM’s formalism analyze competitive contributions from the organization of nodes and edges, and this energy is used to organize or reorganize the network at different levels. This technique can also amplify the important changes in the organization of the network as it goes down to different levels of the organization, capturing the importance of hubs in the network and also at the modular level. Hamiltonian Energy formalism, therefore, proves to be a powerful technique for considering differences in the organization of a network^18^. If ${\Delta HE}_{\theta }={HE}_{\theta }^{[L0]}-{HE}_{\theta }^{[R]}$ is the change in Hamiltonian functions due to removal of key regulators at level $\theta ,$ where ${HE}_{\theta }^{[L0]}$ and ${HE}_{\theta }^{[R]}$ are the Hamiltonian functions for original and removed SARC networks respectively and corresponding community/sub-communities, then we obtain, where ${HE}_{\theta }={HE}_{\theta }^{[R]}$. This demonstrates that removal of KRs causes slight destruction of wiring or rewiring energy that is propagated at all levels of the organization of the SARC network. The relative energy of every key regulators can have at various levels of network organization is shown in Fig. 7.

The Hamiltonian Energy was calculated for hubs with all possible communities in the network at each level. We find that the distribution of energy in the primary SARC network is highest and starts to decrease as the organizational levels increase. The decrease in Hamiltonian Energy indicates the dominance of the interacting edges over the network size, indicating fast processing of information.

Next, in the KRs knockout experiments, we calculated Hamiltonian Energy from the network and communities or sub-communities in terms of understanding the change in energy distributions within the SARC network. Due to KRs knockout, a minor decrease in the Hamiltonian energy is observed at each level (Fig. 7). This means that the elimination of KRs causes a significant loss of wiring or rewiring energy that is propagated across the level of network organization. However, the network does not collapse and maintains the hierarchical features of its organization. This indicates that the network is sensitive to perturbation but tries to maintain its network organization and properties, which are elegantly robust.

### Compactness of network: LCP-DP approach

The LCP architecture not only assists the quick transfer of data through the different network community but also through local processing too. Using LCP approach, we analyzed the SARC network to check its self-organization behavior at different levels of network organization. The LCP-corr of all the communities or sub-communities was measured at different levels presented in Fig. 4b. At each level, the average values of LCP-corr are greater than 0.853 (zero LCP-corr communities aren't taken on average) and these values do not change with the error bar. This means that the network maintains self-organization and compactness and has effective data processing. It serves as a strong dynamic and heterogeneous LCP networks which help in network evolution and reorganization.

### miRNA key regulators network

**Figure 8:**
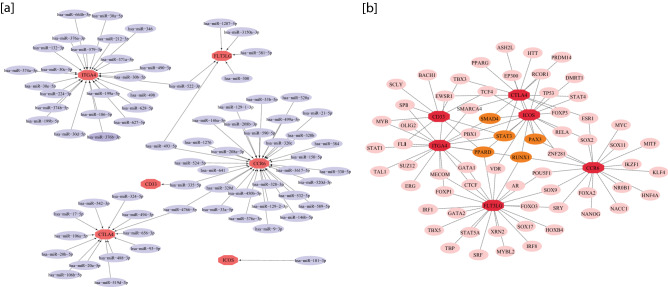
(**a**) Interaction network between key regulators and targeted miRNAs. Orange octagon presented the key regulators and purple circle presented the targeted miRNA. (**b**) The key regulators-transcription factor (TF) regulatory network. Red octagon presented the key regulators, while transcription factors are presented in a pink circle, and highest connection TF is presented in orange circle.

ENCORI was used for screening the key regulator's targeted miRNAs. Seven databases were predicted to identify the miRNAs as the targeted miRNAs of the key regulators. Further, Cytoscape (V 3.6.1) was used to draw the network of the miRNA-key regulator. The resulting network of interactions contains six key regulators and 77 miRNAs, as presented in Fig. 8a. In the Supplementary file, the respective miRNAs targeting key regulators are presented in Table S2.

### TF-key regulators regulatory network

NetworkAnalyst has also enriched TF-gene interactions. ChEA databases were used to predict the TF-KRs interactions. The resulting interaction network consists of 6 key regulators and 65 transcription factors. Furthermore, it has been found that various transcription factors regulator which regulate more than two KRs;, among them, five transcription factors were identified with the highest interaction degree ≥ 3 in the TF-Key regulator’s regulatory network (Table 3). This implies that these transcription factors have strong connections with these key regulators (Fig. 8b). In the Supplementary file, detailed information of transcription factors of key regulators are presented in Table S3.

**Table 3:** The transcription factors of key regulators.

TFs	Genes	Count
RUNX1	CCR6, FLT3LG, ICOS, ITGA4	4
PPARD	CD33, FLT3LG, ICOS, ITGA4	4
STAT3	CD33, CTLA4, ICOS, ITGA4	4
PAX3	CCR6, CTLA4, ICOS	3
SMAD4	CTLA4, ICOS, ITGA4	3

## Discussion

Although some progress in the study of SARC has been made, the exact molecular mechanisms of occurrence and development in SARC are still unclear. Therefore, studying the mechanism to identify the molecular targets for diagnosis and treatment is crucial. In recent decades, the quest for DEGs has been accelerated and its differential expression widely spread.

In this study, the raw gene expression data of six GSE series were obtained from the GEO dataset and a total of 1,126 DEGs were identified, including 270 up-regulated and 856 down-regulated genes that surpassed the cut-off criteria of p-values and fold change. The KEGG pathways results indicate that the down-regulated DEGs were mainly linked with cytokine-cytokine receptor interaction, Tuberculosis, Osteoclast differentiation, pathways in cancer and Human T lymphotropic virus type I infection. In comparison, the up-regulated DEGs were not enriched. These findings also provide helpful evidence for the study of molecular interactions in SARC progression. Indeed, several research studies have indicated that tuberculosis and pathway in cancer are highly associated with the growth and development of SARC. Many studies have been reported a strong association between a history of tuberculosis patients with a higher risk for lung cancer and related mortality. The association between Tuberculosis and the risk of lung cancer in a high-income country was identified in a prospective Korean cohort research study^19^. In patients with a history of lung disease, oxidative stress and local chronic inflammation are mechanisms that increase the risk of lung cancer. Fibrosis is important in the maintenance of inflammation^20–22^. A correlation between SARC and lung cancer has been identified in similar studies^23–26^. In patients with SARC, immunologic defects can result from a lack of immune response against tumors or oncogenic viruses. In comparison, chronic inflammation associated with SARC can contribute to the development of cancer^27^. However, the correlation between Osteoclast differentiation and SARC remains unclear.

**Figure 9:**
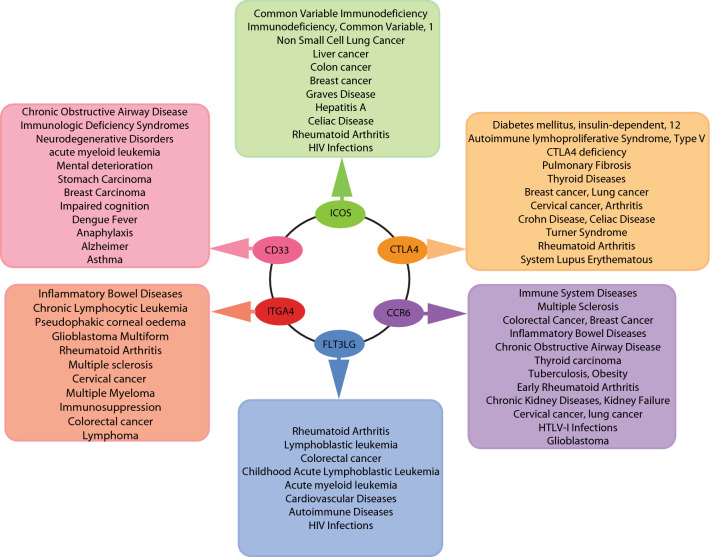
Representation of six key regulators involved directly or indirectly in Sarcoidosis and several other life-threatening diseases, including various types of cancer.

Furthermore, the SARC network was constructed from up and down-regulated genes (DEGs) that gave a network with 877 nodes and 10,546 edges. The constructed network showed hierarchical scale-free behavior, and it means that the network has system-level organizations that involve interconnected communities or sub-communities. Since the nature of the network is hierarchical, each gene activity does not have much importance, but its synchronization shows different significant functional regulations of the network. In the process, individual gene activities assume less significance. In our study, 6 genes out of 877 genes in the network, namely ICOS^**↑**^, CTLA4^**↓**^, GPR29/CCR6^**↓**^, FLT3LG^**↓**^, CD33^**↓**^, and ITGA4^**↓**^ were the most influential key regulators of the SARC network. These key regulators act as the backbone of network activities and its regulations which could be the most probable target gene of disease. Earlier it has been identified that ICOS, CTLA4 and CCR6 polymorphism is related to autoimmune disease risk in patients with Sarcoidosis^28–30^. These key regulators are found to reach the same community and formed a triangular motif till the last level. These genes are also involved in several other diseases which is life-threatening including various type of cancer, Acute Leukemia, Acute Promyelocytic Leukemia, Common variable immune deficiency (CVID), Autoimmune lymphoproliferative syndrome type V, Multiple sclerosis, celiac Disease, Immune system Disease, Crohn’s disease and Alzheimer, etc. presented (Fig. 9).

Our study reported that the gene ICOS is the up-regulated gene in SARC patients compared to healthy controls, as determined by a SARC network analysis. **ICOS** (Inducible T Cell Co-Stimulator) is a co-stimulatory molecule that belongs to the CTLA4 and CD28 cell surface receptor family. Although CD28 is expressed on T cells constitutively to emerge signal for resting T cells to fully activated, ICOS is only up-regulated after activation of cells. A positive signal is provided by this molecule to increase the proliferation of T cells. Studies have been shown that the blocking of ICOS results in the inhibition of immune responses for the T helper type-1, T helper type-2 and T helper type-17^31^. Moreover, recent research has shown that in ICOS-deficient patients, impaired function is observed in CD4 + and CD8 + T cells.

Our finding suggested that the five genes were down-regulated, in which **CTLA4** (cytotoxic T lymphocyte antigen 4) is a member of immunoglobulin’s superfamily, which can inhibit T-cell activation, proliferation and lead to the incidence of peripheral immune tolerance. CTLA4 is a cell surface receptor related to CD28, which binds to CD80 and CD86 ligands. CTLA4 binding to CD86 and CD80 delivers a negative signal to activate T cells by making CD86 and CD80 less accessible to CD28^32^.

The trans-membrane protein **CD33** (Siglec-3) is a sialic acid-binding immunoglobulin like lectin and is expressed in hematopoietic and immune cells. CD33 recognizes glycolipid and glycoprotein. Sialic acid residues have one or more immune-receptor tyrosine based inhibition motif and mediate cell–cell interactions that restrict or inhibit immune responses. The function of CD33 has been involved in many processes such as immune cell growth, immune or malignant cell in adhesion processes, and inhibition of cytokine release by monocytes and endocytosis. However, no studies on CD33 with respect to SARC have been performed. In this study, we found that only one potential miRNA hsa-miR-335-5p that CD33 might target.

The **GPR29** gene that encodes the protein **CCR6** (C–C chemokine receptor type 6) is expressed predominantly in dendritic cells (DC) and memory T cells which is a B cell maturation and differentiation. It is involved in recruiting and migrating DCs and T cells during immunological responses. CCR6 only binds CCL20 and β-defensins.

The **ITGA4** gene encodes a member of the protein family of integrin alpha chains. The ITGA4 integrin family mediates cell–cell adhesions that are particularly important for immune function. Alpha 4 integrin’s are involved in the surveillance, haematopoiesis, inflammation and pathogenesis of cardiovascular diseases. Up-regulation of ITGA4 has been reported in various malignancies in different studies, such as breast cancer, neuroblastoma and melanoma and immune disorders such as Crohn's disease and multiple sclerosis. Down-regulation of ITGA4 and its ligands or inhibition of ITGA4 ligand complex formation was considered a possible therapeutic approach. However, no studies on ITGA4 with respect to SARC have been performed.

**FLT3LG** is a protein-coding gene. DCs provide the key association between innate and adaptive immunity by recognizing pathogens and priming immune responses specific to the pathogen. FLT3LG regulates the production of DCs and is especially essential for the positive classical DCs of plasmacytoid DCs and CD8 and their CD103 positive tissue counterparts. However, there is no report on the correlation between FLT3LG and SARC. We also found that 6 potential miRNA *(*hsa-miR-381-3p, 493-5p, 522-3p, 300, 1287-5p, 3150a-3p*)* that FLT3LG might targeted. SARC is closely related to the immune response. Excessive activation of the immune response to unknown inhaled antigens is considered to be one of the pathogenesis of SARC^33^. Most of the DEGs related to SARC, which we obtained are also related to the immune response. This study believes that the complex relationship of these immune-related DEGs may lead to excessive immune responses.

The network shows fractal nature because of its topological properties, which follow a power-law distribution. It indicates that the network is self-organization and stable. Therefore, the network has a significance of hierarchical properties, and it has no central control system. The KRs knockout experiments show the slight changes in topological properties of the network. However, we did not get a network breakdown, and the network keeps functionally reorganizing itself to stabilize the removal of these key regulators, which is evidence of self-organization. The SARC networks' self-organizing behaviors were also examined by the LCP approach, which leads us to conclude that the network maintains self-organization and is compact with efficient processing of information.

The function of genes is regulated at both transcriptional and post-transcriptional levels. Therefore, we studied the miRNAs-KRs and TFs-KRs networks to provide deeper insights into the regulatory behavior of the identified key regulators. TFs drive gene transcription which may be in a coordinated fashion through genes with associated functions. On the other hand, miRNA are especially powerful regulators of transcript levels at the post-transcriptional level, while it should be observed that there are other less potent and less well-defined categories of non-coding RNAs that also affect transcript levels post-transcriptionally. Thus, we used miRNA and TF targets to identify their targets among the key regulators involved in SARC. In this study we identify some TFs with highest connection with key regulators. RUNX1 is involved in immune response, angiogenesis, embryonic development, hematopoiesis and tumorigenesis^34^. PPARD is a receptor of nuclear hormones which regulates a range of biological processes. It has been suggested that this gene plays a role in the development of many chronic diseases including atherosclerosis, obesity, cancer and diabetes^35^. STAT3 is a transcription factor of cellular signal involved in the regulation of several cellular processes such as cell proliferation, cell differentiation and angiogenesis in normal cells. Diseases like immunodeficiency autoimmunity and cancer are associated with mutations in human STAT3^36^. The PAX3 gene encodes a member of the transcription factors of the paired box or PAX family. During the formation of the skeletal muscle, neural crest derivatives and central nervous system, this protein is expressed and regulates the expression of target genes that impact on differentiation, proliferation, survival and motility in these lineages. PAX3 is also involved in many type of cancers^37^. SMAD4 belongs to the family of signal transduction proteins which are phosphorylated and activated by trans-membrane serine threonine receptor kinases in response to TGF-*β* signaling through many pathways. The function of SMAD4 as a tumor suppressor and inhibits the proliferation of epithelial cells^38^. Our finding showed that these transcription factors formed a linked regulatory network with KRs; therefore, our result signified that the dynamic changes in these transcription factors activities appear in SARC which may play a significant role in regulating the gene function and expression of KRs associated with the appearance and development of SARC.

Therefore, according to this study, the identified few key regulators may act as therapeutic targets for SARC in the future. There are some limitations, such as sample size is limited. In addition, we may not further investigate how KRs-miRNAs networks effects the diagnosis and treatment of SARC in details because of the lack of experimental studies and validations. Despite these limitations, this analysis may provide more accurate results based on the integrated bioinformatics analysis compared to single dataset studies.

## Conclusion

In this study, we performed an integrated analysis based on six microarray gene expression profiles of Sarcoidosis and healthy control to identify DEGs and their associated biological function, and pathways enrichment analysis was performed. The protein interaction network was constructed and analyzed its topological properties and uncovered novel key regulators for Sarcoidosis. Moreover, we constructed miRNA-KRs and TF-KRs network, to provide deeper insight into the regulatory behavior. Our result demonstrated the importance of key regulators and found them to reach the same community and form a triangular motif. All of the genes are known to be involved in immune response and its metabolism. Therefore, these genes and factors are also likely to play a significant role in SARC, considering the preventive impact of immune response on the appearance of this disease. However, the sample size is limited; further studies are also needed to validate the expression and function of the identified key regulators in Sarcoidosis.

## Methodology

### Sarcoidosis associated microarray datasets selection

The NCBI-GEO^39^ dataset is an accessible database that contains gene profiles. Six microarray datasets GSE16538^40^, GSE18781^41^, GSE19314^42^, GSE19976^6^, GSE37912^43^ and GSE75023^44^ were downloaded from GEO datasets^45^*.* In our study, the datasets were selected based on inclusion and exclusion criteria that are (i) Sarcoidosis patient and healthy control studies of humans. (ii) Analysis of gene expression profiling. (iii) Selection of studies with at least six control and six experimental samples. (iv) Excluded datasets if studies without a healthy control. (v) Excluded datasets from other organisms or expression profiling by RT-PCR.

All the datasets and references, which confirmed to the criteria as mentioned above, were manually screened. No ethical approval was required as this study is purely based on bioinformatic analysis.

### Identification of differentially expressed genes

GEO2R^45^ is an online program that allows the comparison and evaluation under the same experimental conditions of two distinct groups of samples. In this study, the selected SARC and healthy control datasets were pre-processed using GEO2R for background correction and normalization. This is based on limma R package^46^. Subsequently, the results of the finding were downloaded in the format of MS Excel, and genes that followed the |logFC (fold change)| ≥ 1 and P-value < 0.05 primary cut-off criteria were considered as DEGs (including regulated genes Up and down). The probes ID without gene annotation or more than one gene annotation were filtered out; the average value of multiple probes corresponding to the same. The probe IDs were converted to gene symbols using Synergizer online server^47^ and the Database for Annotation Visualization and Integrated Discovery (DAVID)^48^.

### Gene ontology and pathway analysis of DEGs

To gain insight into the biological functions and pathways of Up and Down-regulated DEGs were submitted to DAVID online server^48^ was performed to GO classification and Kyoto Encyclopedia of Genes and Genomes (KEGG) pathways analysis^49^. The 10 top entities of the biological process (BP), Cellular component (CC) and molecular function (MF) categories and KEGG pathways were sorted based on P-value. DAVID utilizes Fisher’s exact test to enrich the functions of certain genes. The P-value < 0.05 was considered statistically significant.

### Construction of SARC protein interaction network

The primary SARC PPI network of the identified DEGs was constructed in the Search Tool for the Retrieval of Interacting Genes/Proteins database (STRING)^50^ with an interaction score > 0.40 as the threshold. Through STRING, protein–protein interactions can be investigated and analyzed, the interactions being functional as well as physical associations. These associations are obtained from text-mining, experiment, co-expression analysis, other databases, gene fusion, neighborhood and co-recurrence. Subsequently, in the Cytoscape software (V 3.6.1)^51^ the SARC PPI network was visualized and analyzed.

### Characterization of networks topological properties

The Structural properties of complex networks were described through topological parameter behaviors. The SARC network’s topological properties were computed using the Network Analyzer^52^ and CytoNCA^53^ plugin in Cytoscape. The topological properties analyzed in this study are defined below:

#### Probability of degree distribution

In a PPI network, the degree k represents the number of links the node connects with other nodes. If G = (N, E) describes a graph of a network, where N and E represent the node and edges respectively. The network's degree distribution probability (P(k)) is measured by,

4 $$P(k)=\frac{{n}_{k}}{N}$$

where *nk* = Number of nodes having degree *k* and *N* = Total number of nodes in the network.

P(k) of small world and random network follows Poisson distribution while, for real world, scale free and hierarchical network obeys power-law *P*(*k*) ~ *k*^*−γ*^**,** where, γ is the exponent of degree distribution^54,55^. In hierarchical networks the value of γ becomes close to γ*2.26 (mean-field value) which indicates the importance of community with hubs in the network^13,14^.

#### Clustering coefficients

In a PPI network, the clustering coefficient (C(k)) describes how strongly node neighborhoods are internally connected. This is the ratio of the number of its closest neighborhood edges *e*_*i*_ to the total likely number of edges of degree *k*_*i*_*.* Clustering coefficient *(C(ki))* of i^th^ node for an undirected network can be measured by,

5 $$C({k}_{i})=\frac{2{e}_{i}}{{k}_{i}\left({k}_{i}-1\right)}$$

where e_i_ = Total number of connected pairs among all closest neighbors of the node *i*, k_i_ = degree of the node *i.*

The average clustering coefficient (C(k)) characterizes the entire organization of clusters in the network. Similarly (C(k)), P(k) probably depends on network size. In scale-free networks ***C***(***k***) ~ *constant*, but it obeys power-law in hierarchical network with degree, ***C***(***k***) ~ *k*^−*α*^, with *α* ~ 1, where, *α* is the exponent of Clustering coefficient^13,15^.

#### Neighborhood connectivity

The average connectivity of a node's closest neighbors in a network represents the node's neighborhood connectivity in the network^56^. The neighborhood connectivity is measured by,

6 $${C}_{N}\left(k\right)= {\Sigma }_{q}qP\left(\frac{q}{k}\right)$$

where *P* $\left(\frac{q}{k}\right)$ = conditional probability that a connection belonging to a node with connectivity *k* to another node having *q* connectivity.

In scale free network, *C*_*N*_(*k*) ~ *constant*, while the hierarchical network obeys power-law in degree *k*, *C*_*N*_(*k*) ~ *k*^−*β*^ with *β* ~ 0.5^57^ where, *β* is the exponent of neighborhood connectivity. Furthermore, positive and negative signs in *β* could be an indication of assortivity & dis-assortivity in network topology respectively^58^.

#### Betweenness centrality

Betweenness centrality of a node in a PPI network represents the prominence of information flow through one node to another node through the shortest path^59,60^. The geodesic paths are shown from node i to node j by 'dij(v)' which passes through node 'v' and 'dij'. The Betweenness centrality of a node v can be measured by,

7 $${C}_{B}\left(v\right)={\sum }_{i, j,i\ne j\ne k }\left(\frac{dij \left(v\right)}{ dij}\right)$$

#### Closeness centrality

Closeness centrality represents how quickly information is circulated in the network from one node to another node^61^. The Closeness centrality of the node *i* is described as the reciprocal average length of the geodesic paths between the node and all other nodes connected to it in the network and it is measured by,

8 $${C}_{C}\left(k\right)=\frac{n}{{\sum }_{j}{d}_{ij}}$$

where *dij* = length of the geodesic path between nodes i and j, n = total number of nodes in the network connected to node i.

#### Eigenvector centrality

In a PPI network, Eigenvector centrality of a node *i* (*C*_*E*_(*i*)) in a network is proportional to the sum of closest neighbor centralities^62^, and it is measured by,

9 $${C}_{E}\left(i\right)=\frac{1}{\lambda }\sum_{j=nn(i)}vj$$

where nn(i) = closest-neighbors of nodes i in the network. λ = Eigen value of the eigenvector. v_i_ = ‘Av_i_ = λv_i_’ where A is the adjacency matrix of the network.

The principal eigenvector of A, which corresponds to the maximum positive eigenvalue πmax, represents a centrality score of its eigenvector. Because the eigenvector centrality function of the node varies smoothly across the network and depends on its neighbors, node with high eigen-vector centrality is embedded in the locality of nodes of high eigen-vector centralities, and chance of having isolated nodes in and around the locality is very low^63^. Thus, the centrality of the eigenvector can be used as an indicator of the spreading power of the node in the network.

### Community detection: leading Eigen vector approach

Detecting and characterizing the modular structure and its properties in the hierarchical network are important in identifying network behavior predictions at different levels of hierarchy, as well as accessing the network's organizing principle in the study. In this study, the Leading Eigen Vector (LEV) approach^64,65^ was used in R from the package 'igraph' (http://igraph.sf.net)^66^ to detect the community or modules. The LEV approach is the most effective approach for community detection as it calculates the Eigenvalue for each link, which illustrates the importance of each link, not nodes. We used this approach to detect modules from the primary network, sub-modules from modules at each level of organization, and so on until the motifs level is reached (i.e., 3 nodes and 3 edges), which is the last level of network organization after which the network cannot be further broken. Identifying any sub-module as a community was based on the criterion that it should be found to contain at least one triangular motif (defined by G(3, 3). All the communities, sub-community and sub-sub-community are classified as level-1, level-2 and so on.

### Genes tracing across the networks

In a network, all hubs are important regulators and only those genes which regulate the network from up to down (top to motif level) were considered as the most important and persuasive genes. These genes are termed as ‘Key Regulators’ of the network. To identify these key regulators in the SARC network was done through gene tracing. This gene tracing was conducted up to the level of the motif in different communities or sub-communities obtained from Newman and Girvan’s method of community detection or clustering^65^. Through tracing, the most important and persuasive genes within the network were identified that regulates the network.

### Key regulators knock out experiment

To understand the change in the network organization was observed through the knockout experiment in the absence of these important nodes. We consecutively removed the identified key regulators from the constructed primary SARC network, after that, we measured different topological properties of the reorganized or modified network to study the regulating abilities of these key genes by measuring the degree of structural change due to their absence. Each time we measured the topological properties using Network Analyzer, while in Cytoscape, we used another CytoNCA^53^ plugin for topological properties for Eigenvalue calculation.

### Energy distribution in the network: calculation of Hamiltonian energy

At each level of the network, by following the formalism of the Constant Potts Model (CPM), the Hamiltonian energy (HE) is used as a technique to organize a network at a certain level. HE gives the energy distribution at the global level as well as at the modular level of the network^67,68^. HE of a network and community or sub-community can be calculated by,

10 $${H}^{\left[c\right]}=-\sum_{c}\left[{e}_{c}-\gamma {n}_{c}^{2}\right]$$

where e_c_ = Number of edges in a community *c*. n_c_ = Number of nodes in a community *c* and γ = the resolution parameter acting as edge density threshold which is set to be 0.5.

Further, in the KRs knockout experiment, after removing key regulators from the network, we calculated the HE of network and communities or sub-communities at each level. The difference in HE of the primary SARC network and the key regulators removed SARC network calculated the perturbation caused by the key regulators.

11 $${HE}^{L0}={HE}^{L0}-{HE}_{\theta }^{L0}$$

12 $${HE}^{L1}={HE}^{L1}-{HE}_{\theta }^{L1} and\, so\, on,$$

here L = level in the network, θ = key regulators removed network.

### Compactness of network: local-community-paradigm (LCP) approach

The LCP Decomposition Plot (LCP-DP) is an approach to represent the topological properties of a network in 2D (two dimensional) space of common neighbour’s (CN) index of interacting nodes and local community links (LCL) of each pair of interacting nodes in the network, and it provides number, information of size, and firmness of communities in a network. This can further be used as a measure of self-organization in the network^69^. The LCP correlation (LCP-corr) is the Pearson correlation coefficient between the variables LCL and CN and it is measures as;

13 $${LCP}_{corr}=\frac{cov(CN,LCL)}{{\sigma }_{CN}.{\sigma }_{LCL}} with\, CN>1$$

where c*ov (CN, LCL)* = the covariance between LCL and CN, *σ*_*CN*_ and *σ*_*LCL*_ = standard deviation of LCL and CN.

### miRNA-key regulators network construction

The Encyclopedia of RNA Interactomes (ENCORI) is an accessible web-based tool that focuses mainly on interactions with miRNA targets^70^. ENCORI uses seven developed miRNA target prediction databases, including TargetScan, miRanda, PITA, PicTar, microT, RNA22 and miRmap. In this study, the targeted miRNAs of key regulators were considered miRNAs. Subsequently, this was visualized in Cytoscape software and analyzed the co-expression network of key regulators and their targeted miRNAs^51^.

### TF-key regulators regulatory network

Network Analyst is a comprehensive, accessible web-based tool for network visual analytics of gene expression profiles, statistical meta-analysis and data interpretation^71^. The integrative study of TF-gene interactions for input genes can be supported, and TF’s effect on the functional pathways and expression of the key regulators can be assessed. In this study, TF-KRs interaction was predicted using the ChEA database and Cytoscape software was constructed and visualized the TF-KRs regulatory network.

## Supplementary Information

Supplementary Information 1.

Supplementary Information 2.
