# Supplementary material for: Identification of key regulators in Sarcoidosis through multidimensional systems biological approach
Source: Sci Rep. 2022 Jan 24;12:1236. doi: 10.1038/s41598-022-05129-7 (PMC8786862; doi:10.1038/s41598-022-05129-7)
Supplement: Supplementary file 1 — Supplementary Information 1. [file 41598_2022_5129_MOESM1_ESM.pdf]

Identification of key regulators in Sarcoidosis through multidimensional systems biological approach

Safia Tazyeen[1 2], Mohd Murshad Ahmed[1], Anam Farooqui[1], Aftab Alam[1], Md. Zubbair Malik[2], Mohd Saeed[3], Irfan Ahmad[4 5], Mohammed Abohashrh[6], R. K. Brojen Singh[2*] and Romana Ishrat[1*]

[1]Centre for Interdisciplinary Research in Basic Science, Jamia Millia Islamia, New Delhi-110025, India
[2]School of Computational and Integrative Sciences, Jawaharlal Nehru University, New Delhi-110067, India
[3]Department of Biology, College of Sciences, University of Hail, Hail-2440, Saudi Arabia
[4]Department of Clinical Laboratory Science, College of Applied Medical Sciences, King Khalid University, Abha-61421, Saudi Arabia
[5]Research Center for Advanced Materials Science, King Khalid University, Abha-61421, Saudi Arabia
[6]Department of Basic Medical Sciences, College of Applied Medical Sciences, King Khalid University, Abha-61421, Saudi Arabia

*Co-Corresponding Author:

R. K. Brojen Singh (Associate Professor)

School of Computational and Integrative Sciences,

Jawaharlal Nehru University, New Delhi-110067, India

Email address – brojen@jnu.ac.in

*Corresponding Author:

Dr Romana Ishrat (Associate Professor)

Centre for Interdisciplinary Research in Basic Sciences,

Jamia Millia Islamia, New Delhi-110025, India

Email address – romana05@gmail.com

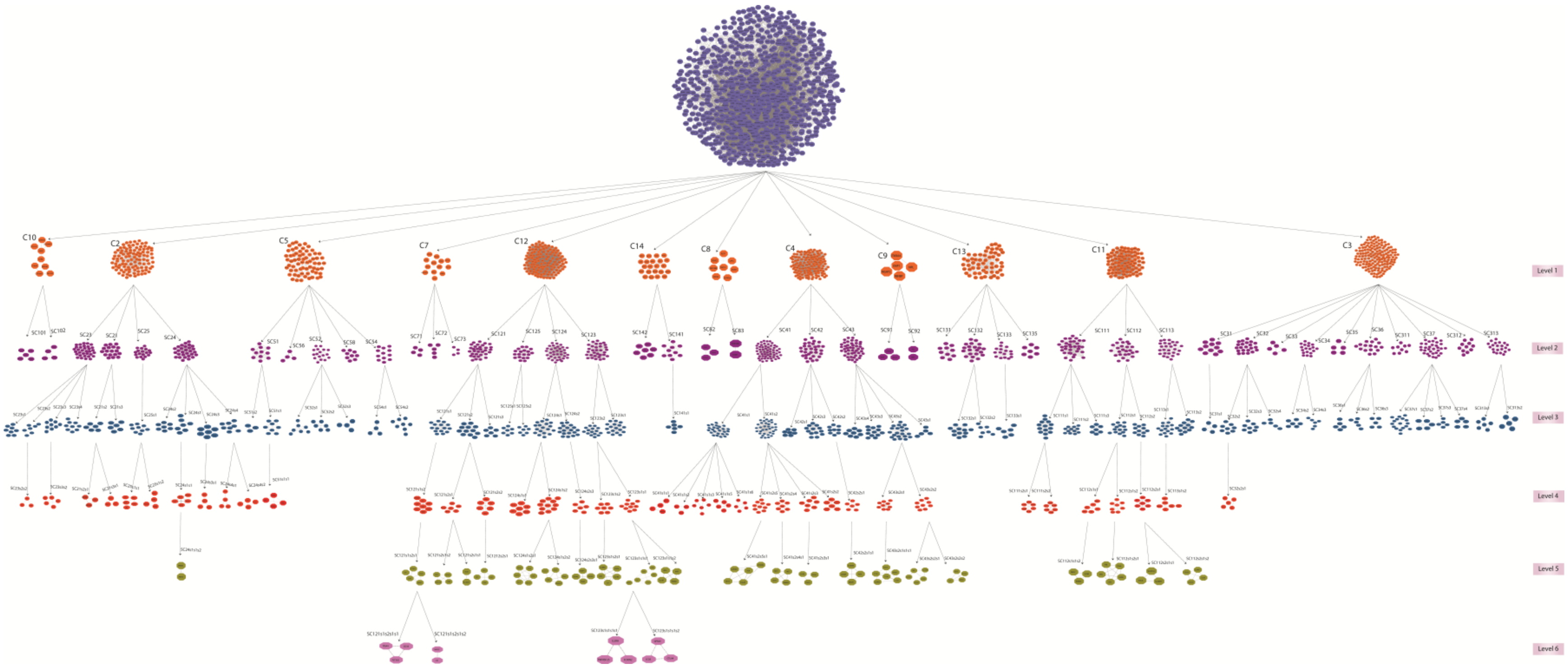

Figure S1: The complete network/communities/sub-communities at six levels accommodates leading hubs and key regulators. The primary sarcoidosis network (purple color) represents level 0. The primary network is divided into thirteen communities after clustering into the next level of organization, i.e., level 1. Each subsequent network represents the next level of the Sarcoidosis network's organization, and arrows indicate sub-communities emerging from the previous communities.
